# Innovation of stamp campaign for strengthening of routine immunization eradication of polio

**DOI:** 10.1186/2047-2994-4-S1-P109

**Published:** 2015-06-16

**Authors:** DJ Sunder, Satti Jewat, Aziz Memon, Bikha Ram Devrajani

**Affiliations:** 1 Medical Research Center LUMHS Jamshoro+RCK Sunders Polio Plus Rotary International, Sindh United(n)Developmental Educational Rural Society, KHIPRO, Pakistan

## Background

Polio is still crippling disease in Pakistan around 306 cases reported by year of 2014, this is alarming situation regarding polio virus around globe too, therefore to strengthen routine immunization eradication of polio is a goal to achieve health standard better regarding morbidity and mortality.

The innovative stamp campaign for psychological, emotionally and healthy activity of stamps on childrens hand about polio to develop interest and impact for polio awareness /education for eradication.

## Aim and object

To eradicate polio free world, and object is educate, aware children about importance of polio drops and routine immunization.

## Material and method

Descriptive study by questioner among the school children to enhance capacity about polio awareness, therefore we put polio stamps on children hands and educate them about polio after that we analysis the data by asking question about polio and its importance on spss version 11.

## Results

Sample size: 500 hundred children were stamping regarding polio.

The outcomes of the properly given answers:

What is polio? Answers were: 353 (70.6%), what happen in polio? Answers were: 293(58.6%)

How many drops given? Answers were: 343 (68.6%), at what age groups taken polio drops? Answers were: 281 (56.2%), Did you like the polio stamp campaign? Answers were: 427 (85.4%)

## Conclusion

Polio is challenging issue by Innovation of stamp campaign play most important role in development according to growth of brain (age) here we are still facing crawling love disease (polio), therefore to regret the wrong myths create awareness engage community and children like polio stamp campaign the outcomes and impact of such campaign gives meaning full evaluation results regarding awareness that will bring change to eradicate polio from globe.

## Disclosure of interest

None declared.

